# Religiosity, Spirituality and Biopsychological Age of Professionals in Russia

**DOI:** 10.3390/ejihpe11040089

**Published:** 2021-10-05

**Authors:** Anna V. Koteneva, Tatiana N. Berezina, Stanislav A. Rybtsov

**Affiliations:** 1 Department of Scientific Basis of Extreme Psychology, Moscow State University of Psychology and Education, 127051 Moscow, Russia; tanberez@list.ru; 2 Center for Regenerative Medicine, University of Edinburgh, Edinburgh EH8 9YL, UK; srybtsov@ed.ac.uk

**Keywords:** biological age, spirituality, psychological age, psychobiological age maturity, religiosity, the rate of aging

## Abstract

The challenges of modern civilization resulted in the premature biological and psychological aging of professionals of working age. This phenomenon raises both medical and psychological problems associated with personality factors that affect psychobiological maturity and the rate of aging. The influence of religiosity and spirituality on biopsychological age remains the least studied area of psychology. Progress in this area will help to identify the components of religiosity—predictors of the aging rate of professionals. The sample included 295 people (148 women) aged 24 to 54 years (average age 31.7 years) and consisted of Christians (67.12%), Muslims (5.76%), Buddhists, deists, Shintoists, etc., (7.79%) and atheists (17.29%). The average work experience was 9 years. Using correlation analysis and methods of multivariate linear regression and *t*-test for independent samples, we found that the religiosity of professionals increases with natural aging and deterioration of their physical condition and does not depend on gender. Religiosity to a greater extent affects psychological age, the indicator of the psychobiological maturity of a professional and, to a lesser extent, biological age. Most of the indicators of religiosity are inherent in a person who is more mature in psychobiological terms. The biological age of professionals increases due to asthenic experiences, while gaining faith in God, unusual religious experiences and the existential meaning of life can reduce it. An increase in the spirituality of professionals is associated with a slowdown in the rate of biological aging.

## 1. Introduction

One of the observed trends in modern society is the premature biological aging of people of working age. Aging is a natural biological process of extinction of the functional activity of organs, cells and systems, which leads to a decrease in the adaptive and regulatory capabilities of a person and increases the risk of diseases and death [1].

Accelerated aging is often accompanied by a deterioration in well-being, the appearance of several psychosomatic symptoms and an increased risk of disability, especially among professionals working under conditions of chronic stress [2,3]. It is extremely important to study the objective and subjective factors that contribute to the maintenance of an optimal functional health status, longevity and durable professional activity.

The reasons for different rates of aging include genetic, epigenetic and stress factors, as well as insufficient individual adaptive potential. Aging research is one of the most important and complex subjects in the life sciences and human health study; therefore, progress in overcoming its underlying challenges can only be achieved through interdisciplinary efforts. While various medical strategies for rejuvenation are focused on the biology of the human body and the metabolic processes, in psychology, emphasis is placed on the study of personal and psychological characteristics that affect the psychosomatic health of people and the rate of individual aging [2,3,4,5,6].

A human is an integral being, including different levels of life activity—physical, mental, spiritual. Religiosity and spirituality are significant characteristics of a person. Religiosity is manifested by faith in God, in the assimilation of religious ideology, values and norms of a particular tradition and participation in cult actions. Signs of religiosity are several objective and subjective indicators that characterize a person’s religious self-identification, their affiliation with a specific religious tradition and the presence of spiritual experience [7]. With a normative type of organization, the spiritual principle has a transforming effect on the soul and body [8]. Therefore, the study of the influence of spirituality and religiosity on the aging rate and biopsychological age are pivotal.

As noted previously, an increasing number of people in old age turn to religion and the spiritual sphere and are involved in spiritual traditions and rituals [7]. A person’s conversion to God is always individual and occurs for various reasons. Some people are brought up in a particular religious tradition, while others acquire faith after finding themselves in a difficult life situation, after experiencing a personal crisis [9].

Spirituality is not always associated with religious tradition. It rather reflects a person’s search for the existential meanings of life, sacred and transcendental, the desire to find the truth and to be guided by moral and ethical norms in interpersonal relationships [8,10]. When a person overcomes difficult life situations, religiosity and spirituality as psychological resources give an understanding of life and death, the aging processes and strength to counteract human aging.

Personal biological age, subjective age, psychobiological maturity and the rate of aging are considered in psychology as manifestations of mental, psychological and somatic health.

In our study, we use the concept of personal biological age—an integral characteristic of age-related changes in the body, all its systems (cardiovascular, respiratory, body balance, metabolic status) and a subjective assessment of health [11].

Usually, biological age is largely determined by the combination of metabolic, structural, functional and regulatory characteristics and adaptive capabilities of the body, and it depends on environmental conditions and lifestyle.

The most important indicator characterizing the rate of age changes is the biological aging index. It is the difference between biological age and expected biological age (the calculated statistical norm for a population). A positive value of the index specifies accelerated aging, while a negative value suggests slower aging [11]. The discrepancy between the chronological and biological age shows the rate of aging and the functional capabilities of the employee at different professional stages and gives hints on the psychophysical health status and performance of the person.

Another important indicator used in our study is subjective psychological age—a person’s assessment of their life path, achievements and prospects on an age scale from 0 to 100 points, where 0 points are the achievements of a newborn child, and 100 points are an old person who has already achieved everything and ends their life. Relative psychological age is the difference between psychological and calendar age, which characterizes the rate of relative psychological aging [11]. Psychobiological maturity is the ratio between the psychological and biological age of a person. Subjective psychological age is a criterion for a person’s psychosomatic and social health, while psychobiological maturity characterizes the correlation of individual psychological and biological capabilities [2,3,11,12].

Numerous studies in psychology have examined the relationship between religiosity/spirituality and psychological, mental and somatic health. However, the obtained results are rather ambiguous. Psychiatrists R. Huguelet and H. G. Koenig note that the religious views of patients have a positive effect on the course of the disease and often act as a beneficial factor in recovery [13]. According to T. Tunkei, as spirituality awakens in HIV/AIDS patients, there is a decrease in the level of depression, hopelessness and anxiety, as well as an improvement in the level of adaptation, satisfaction and quality of life [14]. In a study by J. Carmody et al., the improvement in spiritual well-being was accompanied by a decrease in psychological stress and a decrease in the number of medical symptoms in patients [15].

Among veterans of the US Armed Forces, a high level of religiosity/spirituality contributed to a decrease in the risk of developing lifelong post-traumatic stress disorder, depressive disorder and alcohol consumption and was also associated with increased purposefulness in life [16].

At the same time, a number of authors report on the negative impact of religiosity on mental and somatic health status. Feelings of shame and religious guilt after sinful thoughts or actions can cause concern and worsen well-being [8]. Unfavorable tense relationships between Church members can trigger depression among both clergy and older parishioners. Swinging in faith or feeling insecure about a person’s religious beliefs can be detrimental to mental health.

In modern psychology, the positive influence of religiosity and spirituality on individual psychosomatic health is explained by psychological factors, for example, social support and stress reduction. Z. Zimmer and co-authors believe that religious institutions around the world, such as churches, temples and mosques, are often the centers of interaction, information exchange and social support. The church plays a significant role in the integration of families and the creation of favorable interpersonal relations among single parishioners [8]. In the authors’ opinion, such religious virtues as humility, compassion, gratitude, wisdom and altruism reduce hostility, anger and other negative emotions that negatively affect the psychophysical health status. Internal religious motivation, an active life position and an internal locus of control, prayer and meditation act as means of handling stress. Moreover, religiosity itself, regardless of a person’s confessional affiliation, can have a positive impact on a personality, well-being and behavior [8]. A comparative analysis of adherents of Christianity and Muslims, conducted by H. G. Koenig and co-authors, revealed some similarities in behavior tendencies. In the Western Christian culture and in the countries of the Middle East (including Pakistan, Afghanistan, Malaysia, Egypt, Bangladesh) with a predominantly Muslim population, it turned out that an increase in religiosity is associated with the acquisition of fundamental goals and meanings of life, happiness, optimism, hope and subjective well-being. Religiosity contributed to the growth of self-esteem, the development of an internal locus of control, a decrease in depression and anxiety, changed the attitude towards suicide as a way of solving life problems, prevented the occurrence of suicidal attempts and reduced the attraction to alcohol and drugs [17].

Thus, most of the results reported by many authors show that religious participation is associated with improved mental and social health, healthy behavior and overall physical health [18,19].

Currently, the role of religiosity in maintaining psychosomatic health is being studied insufficiently, and there are no solid data on the unambiguous positive influence of religiosity on the health of professionals. Moreover, according to our best knowledge, there are no publications about the impact of religiosity on the biopsychological age of professionals.

The purpose of this study is to identify specific religious characteristics of professionals that affect their biological age, psychological age, aging rate and psychobiological maturity in different age and gender groups.

We hypothesize that the religiosity and spirituality of professionals are factors that reduce biological age and the rate of aging and, contrariwise, increase their psychological age and psychobiological maturity.

## 2. Materials and Methods

### 2.1. Participants

The sample for empirical research included 295 people (147 men and 148 women)—working in communication technologies, military, psychologists, teachers, managers, lawyers, engineers, programmers, etc., collectively referred in this article as professionals. The term “professional” here means an employee who has a secondary or higher professional education and has practical experience in a distinct licensed field.

An empirical study was carried out in various organizations of the penal system (70 people), law enforcement agencies (60 people), the National Guard of the Russian Federation (60 people), the Russian Pension Fund (15 people), D. Rogachev’s National Medical Research Center for Pediatric Hematology (40 people) and Moscow State Psychological and Pedagogical University (50 people). In each organization, one structural unit was randomly selected (one division, one unit, one department, etc.). Further, all employees of this unit who met the sample selection criteria were surveyed.

Sample selection criteria. (1) The criterion of professionalism: all participants had a secondary or higher professional education and had practical experience in a particular professional field. (2) Age criterion: they all belong to the same age range—from 24 to 54 years. (3) Work experience: all participants had at least 1 year of work experience in the main profession. (4) The criterion of gender equality: the sample included approximately the same number of men and women in each division. (5) The criterion of religious tolerance: the respondents belonged to different spiritual traditions.

The average age of the respondents was 31.7 years (ranging from 24 to 54 years; with the standard deviation is 8.15), and the average work experience was 9 years. Most of the respondents had a higher education (74.6%), and every fourth had a secondary and specialized secondary education (25.6%). Almost half of the participants (48%) were married. The remaining 52% were single or divorced men and women. The sample consisted of followers of the Orthodox tradition—67.12% (198 people), Muslims—5.76% (17 people), Buddhists, Agnostics, Shintoists, Darwinists, Deists—7.79% (23 people) and Atheists—17.29% (51 people), the remaining 2.03% (6 people) preferred not to answer the question about their religious affiliation.

### 2.2. Instruments

To diagnose the spirituality and religiosity of a professional, the following methods were used:

(1) The spirituality scale from the questionnaire “The Resilience of an Adult” by A.V. Makhnach. Such a component of resilience as spirituality evaluates the strength of the spirit, the presence of an existential meaning of life and the level of spiritual and moral development of a person. The Spirituality Scale contains 20 questions. Statistics of internal consistency of the scale divided by the test “Adult Viability”: α Kronbach = 0.937; scale mean = 57.21; variance = 161.16; standard deviation = 12.69; χ2 Friedman between points = 275.99; *p* = 0.001. The reliability of the method has been tested in a series of studies in different countries [20,21].

(2) “Methods for the Study of Religious Activity” by D. O. Smirnov. The “Methods for the Study of Religious Activity” helps to assess the general level of a person’s religious activity and determine the nature of their religious experiences (asthenic, sthenic), the direction of religious motivation (internal or external), the peculiarities of worldview/mentality and the level of involvement in religious practice. The reliability of the method has been tested in studies in the Russian Federation [22].

The method includes six primary scales and two integral indicators. The scale of asthenic experiences (11 questions) is characterized by special feelings for God, which are manifested in the form of shame and guilt, feelings of sinfulness, own inferiority and imperfection. In personal sthenic religious experiences in relation to the Divine, on the contrary, there is a surge of strength and energy, inspiration and delight. The sthenic experience scale consists of 15 questions. The scale of pre-religious experiences includes 10 questions. Pre-religious experiences are inherently not directed towards God but reflect experiences that are often found in altered states of consciousness (unusual bodily sensations, harmony with nature, the world, experience of out-of-body existence). The general indicator of religious experience is a summary indicator of the first three scales and indicates the presence of religious experience and higher transcendental aspirations. The scale of religious motivation characterizes a person’s internal or external orientation in relation to God (11 questions). Internal motivation is a manifestation of the fact that a person puts God at the center of their life, which becomes the meaning of life, and religious traditions allow them to find freedom, to realize themselves in life. External motivation reflects a person’s superficial interest in religion and does not change their intrinsic values and behavior. The worldview/mentality scale (11 questions) measures the level of a person’s religious, theistic or natural science worldview/mentality. The indicator of religious actions characterizes the degree of a person’s involvement in the spiritual tradition and their participation in rituals. The general indicator of religious activity is an integral indicator of all scales that characterizes the general level of a person’s religiosity.

(3) To diagnose indicators of psychobiological age, the methods were developed by K.A. Abulkhanova and T.N. Berezina. It includes two techniques.

‒ Assessment of a person’s biological age, self-assessment of psychological age and an index of psychobiological age [11]. The author of the first technique is V.P. Voitenko [23]. The method calculates the biological age of the individual. For the calculation, objective physiological parameters of the body are used, including arterial systolic pressure, arterial diastolic pressure, duration of breath-holding after a deep breath (in men), body weight (for women), static balancing on the left leg (without training), as well as subjective indicators—(subjective) self-assessment of health (28 questions about the presence of psychosomatic symptoms). The expected biological age (the statistical norm for a given age), as well as the rate of aging of the organism (the difference between biological age and the expected biological age) are also calculated. In psychological science, there are different criteria for determining the rate of aging [2; 3]. In our study, we used the criteria for the aging rate according to L.M. Belozerova. The difference between biological age and expected biological age in the range from −15 to −5 characterizes a slower rate of aging; within the range from −4.99 to +4.99 is the natural rate of aging of the body; the difference from +5 to +15 is premature aging. The validity of the method has been verified in a series of studies in Russia [24], as well as in cross-cultural studies [11]. The authors assessed the structural reliability of the biological age method by Voitenko. They calculated the correlation coefficient between the variables that are part of the methodology. They showed a good correlation between all the indicators, which indicates the structural reliability of the test [25]. Before we started our study, we assessed the retest reliability of the biological age (BA) measurement method using the Pearson correlation coefficient. The indicator was re-measured six months later (the entire study was conducted in 2019). For women in the age group up to 35 years, the retest reliability of the method = 0.69. For men in the age group up to 35 years, the retest reliability of the method = 0.62.
‒ The second method, “self-assessment of psychological age on a 100-point scale”, was proposed by T.N. Berezina [11]. The assessment of the adequacy of age perception was based on the criteria given in the work of O.Yu. Strizhitskaya. The discrepancy between psychological and calendar ages ranging from −4 to +4 years is normal; indicators outside this interval indicate the inadequacy of the perception of their psychological age and often the presence of a neurotic state [2; 3]. The indicator of psychobiological age maturity (the ratio between biological and psychological age) provides information on how much the respondent feels younger or psychologically older than their biological capabilities. We carried out a preliminary comparison of our method with the known method for evaluating subjective time [26], which uses a scale similar to our 100-point scale. The results on the Barack total subjective age scale largely corresponded to the results of the assessment of the subjective personal age by our method (according to the Pearson correlation coefficient). Before starting our study, we assessed the reliability of the method for measuring psychological age (PA) using the Pearson correlation coefficient. The indicator was re-measured six months later (the entire study was conducted in 2019). For women in the age group up to 35 years, the retest reliability of the method = 0.50. For men in the age group up to 35 years, the retest reliability of the method = 0.30.

### 2.3. Procedure

The work was carried out during 2019–2020 with the financial support of the Russian Science Foundation No. 19-18-00058. The research program was approved by the Ethical Commission of Psychological and Interdisciplinary Research of the Faculty of “Extreme Psychology” of the Moscow State Psychological and Pedagogical University (Moscow, Russia), protocol No. 59k-03/19 of 23 April 2019 and is compiled in accordance with the rules of the 1975 Declaration of Helsinki (as amended in 2013) and is also guided by the ethical code of psychologists when conducting psychological research. A package of questionnaires was prepared for each participant, which was filled out in the group. A measurement of objective indicators of biological age was carried out individually. All studies were conducted anonymously. Participants gave written voluntary informed consent to participate in the study.

### 2.4. Analysis

Statistical data processing was carried out using the SPSS statistic program (*t*-test for independent samples, correlation analysis, multivariate linear regression method, checking the normality of the distribution). At the beginning, the variables were tested for normal distribution using the Kolmogorov–Smirnov criterion. Normal distribution was confirmed for all key indicators of our study: index of relative biological aging, index of relative psychological aging, psychobiological maturity and indicators of religiosity.

At the next step, we analyzed descriptive statistics of indicators of religiosity and biopsychological age both for the entire sample and for samples of male and female workers. Given the normal distribution of variables for comparative analysis, we used the *t*-test for independent samples. This criterion allows to identify significant differences in the studied scales between professionals of different sex. The significance level of the differences that was considered was *p* = 0.05 or *p* < 0.05. The same criterion was used for the comparative analysis of indicators between the two age groups. Correlation analysis with the calculation of Pearson’s correlation coefficients allowed us to reveal the presence or absence of connections between the indicators of religiosity and the biopsychological age of professionals. The significance level of the correlation coefficients that was considered was *p* = 0.05 or *p* < 0.05. At the final stage, to determine the religious characteristics that affect biological age, psychological age, self-assessment of the psychosomatic state, the rate of aging and multiple linear analysis were used with a step-by-step exclusion method. The independent variables were the indicators of religiosity and spirituality: asthenic experiences, sthenic experiences, pre-religious experiences, general indicator of religious experience, religious motivation, worldview/mentality, religious actions, religious activity and spirituality. Dependent variables are indicators of biopsychological age: self-assessment of health, biological age, expected biological age, relative biological aging index, psychological age, psychobiological age maturity, relative psychological aging index and calendar age. The correlation matrix between the dependent and independent variables is reflected in the correlation heatmap. The stepwise test is based on the probability of F-inclusion ≤50, F-exclusion ≥100. To evaluate the regression model, the following indicators were calculated: R-squared, non-standardized coefficient B, standardized coefficient Beta, constant, confidence interval and significance level.

## 3. Results

The results of the study are presented in Table 1. All indicators of religiosity correspond to the average level for the entire sample. The biological age of professionals exceeds the calendar and expected biological age. The relative biological aging index reflects the approximate correspondence of biological age and expected biological age. Some significant differences in the T-criterion for independent samples were found between men and women. It turned out that women significantly differ from men only in one indicator of pre-religious experiences: they are more likely to experience an altered state of consciousness. There are no differences in all other manifestations of religiosity. Religiosity does not depend on biological gender; it is the basis of human existence as such. While there are significant differences in the indicators of biopsychological age. Female professionals are twice as likely to note somatic signs of health disorders, but with the same calendar age, their biological age and the rate of biological aging are much lower. In male professionals, the rate of aging corresponds to an accelerated rate, and in women—to the usual physiological rate of aging of the body. Although the indicators of psychobiological age maturity in all professionals correspond to an adequate level, women show greater age maturity. At the same time, according to the calendar age, men (average age 32 years) and women (average age 31 years) do not differ significantly.

The dynamics of religiosity and biopsychological age indicators correlate with professional activity. In accordance with the periodization adopted by the International Symposium on Age Periodization in 1965, two groups of middle-aged professionals were identified. The first group included employees aged 22 to 35 years (the average age is 27.5 years), and the second group—36 to 54 years (the average age is 41.7 years). The results are shown in Table 2.

The differences between the groups were revealed by three indicators of religiosity—sthenic experiences, religious actions and spirituality. As people grow up naturally, when they turn to God, they often feel a surge of strength, inspiration and support from God. This becomes a motive for the life transformation, when religious traditions and rituals acquire special significance. The acquisition of existential, spiritual and moral values at a more mature age ensures the entry of a person into a spiritual tradition. There were no significant differences in other religious characteristics. With the calendar age, not only the inner spiritual life changes, but also the somatic health, biological and psychological age, psychobiological age maturity, and the rate of biological aging.

In the second age group, somatic health is assessed as deteriorated, the biological age is 3 years higher compared to the first group, but the rate of biological aging corresponds to the usual rate of aging. While younger professionals have a tendency to accelerated aging, depletion of physical resources of the body, a decrease in the functional, regulatory and adaptive capabilities of the body. Professionals of two groups evaluate themselves older than their calendar age, and young people inadequately estimate their age, in contrast to more personally and professionally mature people.

The degree of religiosity affects the characteristics of the biopsychological age of a professional. In this study, a low level of religiosity is observed in 24.75% of professionals. This group is heterogeneous in its worldview. Half of its representatives (50.0%) are atheists who reject the religious picture of the world, 43.2% of the respondents formally belong to Christianity, Islam or Buddhism. On the one hand, they identify themselves with one or another religion; on the other hand, they do not show any religious activity. Out of the sample of professionals, 6.8% did not answer the question about religion.

The middle level of religiosity was found in 46.44% of respondents. This group of professionals also includes atheists (9.5%) who have undergone unusual, spiritual experiences, which, however, did not significantly affect their worldview/mentality and religious activity. All the other representatives of the group attributed themselves to one or another tradition. From a psychological point of view, the middle level of religious activity means a state of “warm coolness” in relation to faith and religious practice. In their spiritual life, they encounter religious experiences, believe in God, occasionally participate in rituals and identify themselves as belonging to a particular spiritual tradition. However, religious faith does not radically affect the way of life, and it does not become its dominant.

A high level of religious activity is characteristic of 28.81%. This group consists exclusively of adherents of the religious tradition. Their spiritual life is highly filled not only with a variety of religious experiences, but it also differs in a formed religious worldview/mentality, the presence of higher aspirations to God and compliance with established religious norms and rituals in their daily life.

**Figure 1:**
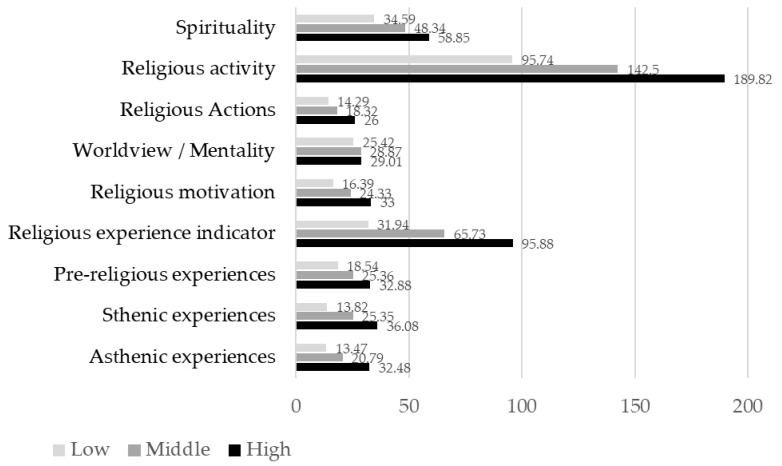
Religiosity indicators in groups of professionals with different religiosity levels (low, middle and high). The severity of the indicator of religiosity in points (*Y*-axis) for different indicators of religiosity (*X*-axis). Sample size: low religiosity (73 people), middle (137 people) and high (85 people). The level of religiosity is indicated by a grayscale.

**Figure 2:**
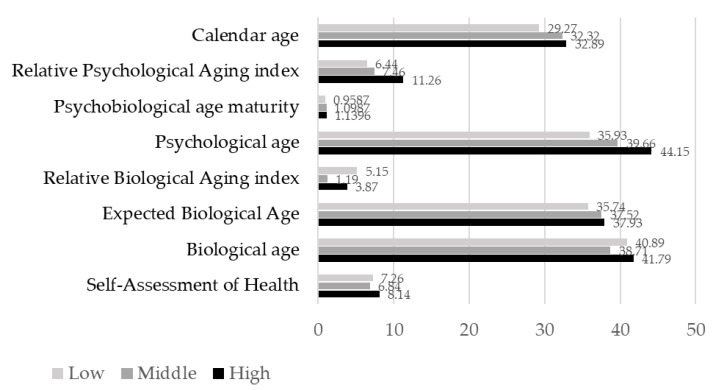
The biopsychological age indicators of professionals with different religiosity levels (low, middle and high). The biopsychological age indicators of religiosity in points (*Y*-axis) for different indicators of biopsychological age (*X*-axis). Low religiosity (73 people), middle (137 people) and high (85 people). The level of religiosity is indicated by a grayscale.

Figure 1 illustrates the data on religious indicators in three groups, and Figure 2 shows the indicators of the biopsychological age of professionals with different levels of religiosity.

For all indicators of religiosity, significant differences between the groups were revealed using the t-test for independent samples (*p* < 0.001). Immersion in the religious tradition is accompanied by the growth of religious spirituality in a person, the acquisition of an existential meaning of life and the desire to be guided by spiritual and moral norms of behavior in interpersonal relations.

When comparing the indicators of the psychological age of professionals with different levels of religious activity, it turned out that there are no significant differences in the self-assessment of their somatic health. People are equally attentive to their health, regardless of the degree of immersion in religious life.

The biological age of professionals with a high level of religiosity is higher than that of professionals with a middle level of religiosity (T_emp_. = 2.191; *p* < 0.05). The expected biological age and calendar age is also higher for professionals with a middle (T_emp_. = 2.319; *p* < 0.05) and a high level of religiosity (T_emp_. = 2.685; *p* < 0.01) compared to professionals with a low level of religiosity. Differences were found between the rates of biological aging between professionals with a middle and low level of religiosity (T_emp_. = 2.721; *p* < 0.01), as well as between professionals with a middle and high level of religiosity (T_emp_. = 2.045; *p* < 0.05). At the same time, the rate of aging is the highest among non-religious professionals. Psychological age, psychobiological age maturity and the index of psychological aging increase with an increase in the level of religiosity, but significant differences are achieved only when comparing groups with low and high levels of religiosity (respectively, T_emp_. = 3.182; *p* < 0.01; T_emp_. = 2.080; *p* < 0.05; T_emp_. = 1.925; *p* < 0.05).

The correlation analysis revealed significant, but not stable, relationships between the following religious characteristics of a professional and indicators of biopsychological age:

Asthenic experiences and self-assessment of health (r = 0.101), biological age (r = 0.125), expected biological age (r = 0.149), psychological age (r = 0.150) and calendar age (r = 0.149). The feeling of guilt before God and the feeling of sinfulness are accompanied by a deterioration in somatic health, an increase in biological, psychological and calendar age and corresponds to the age group.

Sthenic experiences and psychological age (r = 0.202), psychobiological age maturity (r = 0.132), relative psychological aging index (r = 0.117), expected biological age (r = 0.180) and calendar age (r = 0.192). The surge of strength and inspiration associated with turning to God are observed in people as they naturally grow up. People who are distinguished by personal maturity feel psychologically older than their peers, and sometimes, they do not always adequately feel and accept their age.

Pre-religious experiences and self-assessment of health (r = 0.214), psychological age (r = 0.100) and calendar age (r = 0.123). Unusual experiences associated with the entry of a person into an altered state of consciousness are correlated with a deterioration in well-being and an increase in both the calendar and psychological age.

General indicator of religious experience and expected biological age (r = 0.113), psychological age (r = 0.156), psychobiological maturity (r = 0.097) and relative psychological aging index (r = 0.118). The deeper a person’s religious experience, the closer their biological age indicators are to the population ones, the more pronounced their age maturity, subjective perception of their age and psychological aging.

Religious motivation and psychological age (r = 0.152), psychobiological age maturity (r = 0.100), relative biological aging index (r = 0.110) and expected biological age (r = 0.097). The desire for an inner religious life is noted among professionals who feel personally and psychologically more mature. As the religious intrinsic motivation raises, there is an increase in both psychological age and age maturity and an acceleration in the rate of biological aging.

Religious actions and psychological age (r = 0.153), calendar age (r = 0.216) and expected biological age (r = 0.227). Involvement in religious practice increases with the natural and psychological maturation.

Religious activity and psychological age (r = 0.159), psychobiological age maturity (r = 0.095), relative psychological aging index (r = 0.105) and expected biological age (r= 0.113). The general level of religiosity also increases with the psychological age.

Spirituality and the relative biological aging index (r= −0.102), psychological age (r = 0.168), psychobiological age maturity (r = 0.143), calendar age (r = 0.159) and expected biological age (r = 0.130). The acquisition of existential meanings and the desire to be guided by spiritual and moral values in everyday life are associated precisely with the psychological maturity and are also accompanied by a slowdown in biological aging.

**Figure 3:**
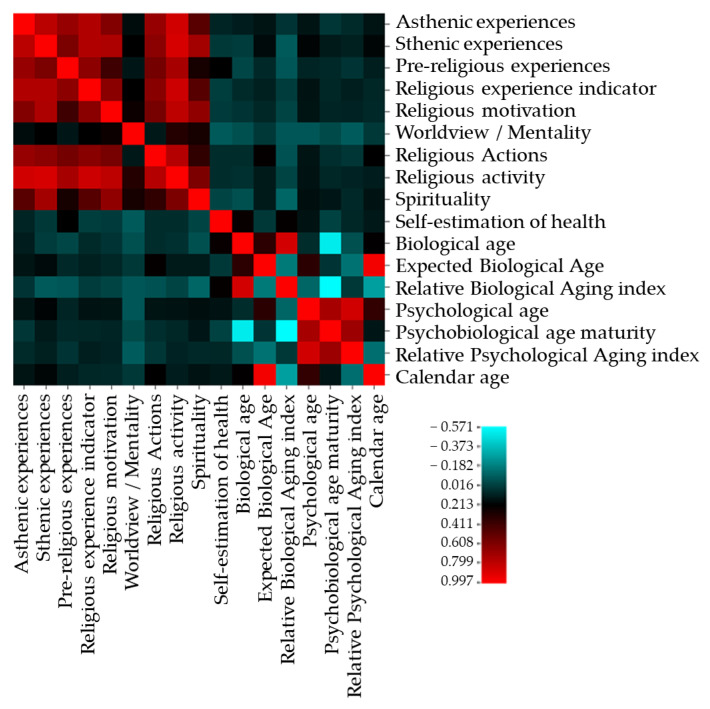
The correlation matrix of the independent and dependent variables included in the regression analysis.

To identify religious components that can influence the self-assessment of health, biological age, psychological age and the rate of aging, a regression multiple analysis was also performed by the step-by-step inclusion method (Figure 3).

The characteristics of religiosity and spirituality were used as independent variables. The study revealed a difference between the genders only in terms of the scale of pre-religious experiences, and in terms of age—in three indicators: sthenic experiences, religious actions and spirituality. However, we were primarily interested in religious predictors of the biopsychological age of professionals. Therefore, gender and age variables were excluded from the independent variables. As our previous studies show, these variables can affect certain components of biopsychological age—biological age, self-assessment of health and the rate of aging in employees of the penal system [2].

The coefficient of multiple determination appeared to be insignificant, explaining to a very small extent the proportion of variance of the dependent variable. That is to say, it cannot be assumed that only religious factors are predictive in relation to biological age, self-assessment of health, psychological age, psychobiological maturity and the rate of biological aging. However, the values of the coefficients of the identified predictors are significant, which allows us to consider these factors as possible reasons that affect the indicators of the biopsychological age. The results are presented in Table 3.

Based on the results obtained, the following regression equations can be created.

Biological age = 0.361 × asthenic experiences − 0.246 × pre-religious experiences − 0.083 × spirituality + 42.411.

Asthenic experiences increase biological age (coefficient 0.361, with a 95% confidence interval of 0.261 to 0.461). Biological age is reduced by pre-religious experiences (coefficient −0.246, 95% confidence interval from −0.354 to −0.138) and spirituality (coefficient −0.083, confidence interval from −0.125 to −0.041). The constant is positive; its coefficient is 42.411 (95% confidence interval is from 39.933 to 44.889).

Self-assessment of health = 0.251 × pre-religious experiences − 0.037 × general indicator of religious experience + 3.261.

Self-assessment of health is increased by pre-religious experiences (coefficient 0.251, 95% confidence interval from 0.197 to 0.305). Self-assessment of health decreases by general indicator of religious experiences (coefficient −0.037, confidence interval from −0.05 to −0.024). The constant is positive; its coefficient is 3.261; the confidence interval is from 2.165 to 4.357.

Psychological age = 0.329 × sthenic experiences + 31.618.

Psychological age is increased by sthenic experiences (coefficient 0.329, 95% confidence interval from 0.236 to 0.422). The constant is positive; its coefficient is 31.618; the confidence interval is from 29.057 to 34.179.

Psychobiological age maturity = 0.005 × spirituality + 0.841.

Psychobiological age maturity is increased by spirituality (coefficient 0.005, 95% confidence interval 0.003 to 0.007). The constant is positive; its coefficient is 0.841; the confidence interval is from 0.741 to 0.941.

Relative psychological aging index = 0.060 × general indicator of religious experiences + 4.311.

The relative psychological aging index is increased by the general indicator of religious experiences (coefficient 0.060, 95% confidence interval from 0.030 to 0.090). The constant is positive; its coefficient is 4.311; the confidence interval is from 2.144 to 6.478.

Of all the religious characteristics, asthenic, sthenic, pre-religious and religious experiences in general and spirituality act as predictors. The biological age of a professional is increased by asthenic experiences, namely, a sense of guilt and sinfulness before God. Whereas the acquisition of faith in God and existential meaning in life, reliance on spiritual and moral values, as well as the presence of unusual religious experiences, can reduce it. The experience of entering into altered states of consciousness impairs somatic and mental well-being. However, religious feelings themselves improve the subjective health status. Sthenic religious experiences, when a person has a dialogue with God, experiencing a surge of strength and energy, feeling inspiration and delight, act as prognostic sign of greater psychological age. Such communication enriches a person, changes the attitude towards life, towards oneself and towards others. Spirituality increases the psychobiological maturity of the professional. Intense religious experiences may accelerate psychological aging. In some cases, this can manifest itself in the acquisition of wisdom; in other cases, it can lead to an inadequate appreciation of a person’s calendar age.

## 4. Discussion

### 4.1. Discussion of the Main Results

The hypothesis of the study was partially confirmed. The religiosity of a person is largely associated with psychological and age characteristics and to a lesser extent with biological characteristics.

The first part of the hypothesis—“religiosity and spirituality of professionals are factors that reduce biological age and the rate of aging”—was partially confirmed, and the results are contradictory.

The study demonstrated that asthenic experiences could increase the biological age of a professional and worsen their health (the self-assessment of health indicator increases), which is indirectly confirmed by the data provided by Z. Zimmer about the deterioration of well-being and an increase in the level of anxiety due to a sense of shame and guilt before God. Religious doubt or a feeling of uncertainty about religious beliefs led to negative consequences for mental health [7]. The review of F. M. Shan’kov [9] also provides similar data indicating that negative religious experiences (anger at God, a sense of sinfulness, a sense of abandonment by God, etc.) and negative religious handling impair physical health, well-being and the immune system and increase pain.

Pre-religious experiences also impair the worker’s well-being. Religious motivation increases the relative biological aging index. No other indicators of religiosity have significant correlations with indicators of biological age and the rate of biological aging. Moreover, most of the indicators of religiosity (asthenic experiences, sthenic experiences, pre-religious experiences, general indicator of religious experience, religious motivation, religious actions, religious activity) turned out to be closely related to either calendar age or expected biological age.

The calendar age of the professional plays an important role. The older people are, the more often they turn to religion. Problems that arise in various spheres of a person’s life (spiritual, professional, family, social and health) often become the reason for their conversion to God, evangelizing and churching. The need for religious experiences and participation in spiritual practice are increased in people who face damages of their somatic health, with signs of biological and psychological aging. F. M. Shan’kov [9] provides data confirming our results: when physical health worsens, people often resort to prayer, meditation and rituals in order to improve their well-being and heal.

That is to say, the religiosity of workers increases with age, and this growth reflects the natural tendencies characteristic of the expected biological age of a particular age group. This suggests that the older workers become, the more often they return to the religious tradition within which their religious identity and worldview were formed.

The results of the correlation analysis indicate that the rate of biological aging slows down with the growth of human spirituality. Spirituality does not necessarily mean belonging to a particular religious tradition, although it often manifests itself in the bosom of a particular religious practice. The search for truth, the acquisition of the highest absolute values and eternal meanings of life give ontological confidence to a person. Spiritual and moral values and moral norms not only regulate interpersonal relations, but also become a way of coping with difficult life situations. Finding the meaning of life and understanding the goals of a human can change one’s attitude towards one’s own aging and death. The data obtained coincide with the results of the study [2], showing that spirituality slows down the rate of biological aging in law enforcement officers.

Positive spiritual experience that a person finds in the search for God and finding the meaning of life can slow down the natural processes of physical exhaustion of the body.

The second part of the hypothesis—“the religiosity and spirituality of professionals are factors that increase their psychological age and psychobiological maturity”—has been fully confirmed. According to the results of the correlation analysis, asthenic experiences, pre-religious experiences, general indicator of religious experience, religious motivation, religious actions, religious activity and spirituality increase with increasing subjective age. The presence of the experience of communion with God, in which a person feels the support of God, an influx of spiritual and physical strength, spiritual experience in general and the need for religious experiences make a person psychologically older than their calendar age. Reliance on spiritual and moral norms of behavior in interpersonal relationships as well as striving for spiritual, existential values give a person additional psychological opportunities in comparison with non-religious colleagues with the same biological age. Certain religious indicators such as sthenic experiences, general indicator of religious experience and religious activity play a role in psychological aging. On the one hand, an increase in psychobiological age maturity and relative psychological aging index may indicate the worker’s life wisdom. On the other hand, in the case of excessively high indicators, it serves as a signal of psychological exhaustion, and when combined with other symptoms, for example, a reduction in professional achievements, as a signal of depersonalization, even emotional burnout.

Regression analysis allowed us to identify religious predictors of almost all indicators of the worker’s biopsychological age. Of all the religious characteristics, attention is drawn to asthenic and sthenic experiences, the presence of religious and pre-religious experiences in general, as well as the spirituality of a person. They act as predictors of biological age, psychological age, psychobiological maturity, self-assessment of health and the rate of aging. That is to say, the real experience of being God-forsaken and communion with God play a greater role in aging processes than just a religious picture of the world and formal religious identity.

Despite the differences in the understanding of the goals of religious life, a comparison of Buddhist and Christian elderly in Singapore showed that religion plays an important role in positive adaptation to the physical, social and existential processes of aging [27].

However, the revealed components of religiosity are not the only factors of biopsychological age. Along with them, other personal qualities also have a great influence on the rate of aging. Several studies have found that the improvement in the well-being of the penitentiary system workers improves with the growth of spirituality and meaningfulness of life, the presence of fundamental life goals, emotional saturation of life, satisfaction with self-realization and self-confidence. The rate of their biological aging is influenced by life goals and locus of control–life. Having fundamental goals that make life meaningful slows down the rate of aging, while persuasion and desire to control your life in all its manifestations accelerates biological aging [2]. The biological age of law enforcement officers is reduced by such psychological resources as self-efficacy, perseverance, an internal locus of control, constructive coping strategies and family support. Psychological age and psychobiological age maturity also increase with the actualization of such resources as self-efficacy, internal locus of control and resilience [3].

There are also a number of limitations in the dissemination of the results obtained, due to the restrictions in both the methods used and the sample studied.

### 4.2. Evaluation of Data and Methods Limitations

The methods used to diagnose religious characteristics give a general idea of the severity of several formal indicators. They are not intended to assess the knowledge of dogmas, foundations of doctrine, spiritual and moral norms that reflect the specifics of spiritual practice. We left outside the framework of the analysis such questions as the depth of faith, prayer, repentance and the inner work of a religious person over their mistakes, such as sinful thoughts, feelings, experiences and actions. Although, it is these aspects that characterize the content side of a person’s spiritual life and their spiritual health [8].

In this study, we were interested in the very phenomenon of religiosity in terms of experiences, worldview, motivation and participation in ceremonies and rituals. Although we are aware that in different traditions, religious manifestations have significant qualitative differences.

Probably, conducting a study on representatives of one confession would allow us to interpret the results more deeply, considering the specific practical experience of the spiritual tradition.

The study was conducted based on various state organizations in Moscow and covered working professionals aged from 24 to 54 years. All the subjects had higher or secondary and special secondary education and professed to be Christians, Muslims, Buddhists, Deists, Shintoists, as well as atheists. Conclusions about the influence of religiosity on biopsychological age do not apply to unemployed retirees and young people who do not have an education and professional experience, as well as adherents of other religious traditions. Further studies may be needed to reveal whether this result can indeed be widespread to the other age groups and representatives of other religious confessions.

### 4.3. Impact and Perspectives

We believe these results have important practical value. Religiosity is one of the resources that a person can use in life. Psychological support of employees at different stages of professional development should consider the peculiarities of their religiosity to avoid emotional burnout and impairment of psychosomatic health, prevent premature biological aging and form psychological maturity. It is advisable to include specifically developed training programs in the psychological training of employees, aimed not only at reducing negative functional states, but also at increasing the meaningfulness of life, spirituality and the formation of fundamental life goals. This can be useful for both religious and atheistic professionals.

## 5. Conclusions

Religiosity is an important characteristic of a person’s existence; it does not depend on gender and age. The ambiguous influence of religiosity on the health status can be explained by the fact that the goal of religion is not to improve psychosomatic health as such. Rather, it is about the spiritual health of the individual that reveals the quality of a person’s relationship with God and the people around. The ideal state of this health is holiness. Health is chastity, the holistic wisdom that characterizes a person from the point of view of their spiritual and mental–bodily purity—the highest state of moral perfection [8].

For example, in the Orthodox Christian tradition, the concepts of health and illness are associated with understanding the meaning and goals of human existence, the main of which are the salvation of the soul and the spiritual transformation of the individual.

The attitude of a Christian to secondary goals (achieving good somatic health, self-realization, inner harmony, etc.) is based on whether they help to gain immortality or doom a person to eternal death [8]. Ideally, if a person is faced with a choice of saving their soul or bodily health, they would choose the first. At the same time, the acquisition of existential meaning in life can slow down the pace of biological aging, and the feeling of joy of communion with God can lower the biological age.

## Figures and Tables

**Table 1:** Indicators of religiosity and biopsychological age of professionals and the significance of their differences according to the T-criterion between men and women.

Indicators	Total Sample (295 People)		Men (147 People)		Women (148 People)		T _emp_.	P—Significance Level
	M ^1^	SD	M	SD	M	SD		
Asthenic experiences	22.35	8.91	22.64	9.09	22.06	8.74	0.557	0.578
Sthenic experiences	25.59	10.28	25.64	9.90	25.54	10.67	0.084	0.933
Pre-religious experiences	25.84	7.56	25.01	8.06	26.67	6.97	−1.896	0.050
General indicator of religious experience	66.05	30.41	69.32	28.81	62.81	31.69	1.848	0.066
Religious motivation	24.86	8.41	25.27	8.14	24.47	8.68	0.816	0.415
Worldview/mentality	28.06	4.44	28.07	3.48	28.05	5.23	0.040	0.968
Religious actions	19.54	6.46	20.08	6.65	18.99	6.24	1.449	0.148
Religious activity	144.57	38.09	145.66	37.60	143.48	38.67	0.493	0.623
Spirituality	47.97	16.27	47.07	16.11	48.85	16.45	−0.937	0.349
Self-assessment of health ^2^	7.32	5.45	5.38	4.91	9.24	5.29	−6.495	0.001
Biological age	40.14	10.35	45.70	8.29	34.62	9.18	10.88	0.001
Expected biological age	37.19	5.23	38.93	5.25	35.47	4.61	6.010	0.001
Relative biological aging index	2.94	9.83	6.77	8.62	−0.86	9.51	7.222	0.001
Psychological age	40.03	16.68	40.22	15.88	39.84	17.49	0.199	0.843
Psychobiological age maturity	1.08	0.56	0.92	0.39	1.23	0.645	−4.990	0.001
Relative psychological aging index	8.30	15.63	7.63	15.42	8.97	15.85	−0.740	0.460
Calendar age	31.73	8.15	32.48	8.39	30.99	7.86	1.580	0.115

^1^ M—average value; SD—standard deviation. ^2^ According to the authors of the method, the higher the indicator, the worse the health.

**Table 2:** Indicators of religiosity and biopsychological age of professionals and the significance of their differences according to the T-criterion in different age groups.

Indicators	Age Group 22–35 Years (208 People)		Age Group 36–54 Years (87 People)		T _emp_.	P—Significance Level
	M	SD	M	SD		
Asthenic experiences	21.84	9.19	23.57	8.10	−1.613	0.127
Sthenic experiences	24.62	10.32	27.90	9.84	−2.575	0.010
Pre-religious experiences	25.39	7.70	26.91	7.16	−1.613	0.119
General indicator of religious experience	65.25	30.76	67.97	29.65	−0.710	0.485
Religious motivation	24.38	8.55	26.01	7.99	−1.562	0.130
Worldview/mentality	27.98	4.75	28.24	3.59	−0.514	0.646
Religious actions	18.95	6.53	20.94	6.11	−2.506	0.010
Religious activity	142.34	38.72	149.89	36.18	−1.602	0.120
Spirituality	46.43	15.69	51.64	17.14	−2.443	0.050
Self-assessment of health	6.77	5.11	8.63	6.02	−2.530	0.010
Biological age	39.08	9.95	42.67	10.91	−2.641	0.010
Expected biological age	34.66	3.24	43.27	3.89	−18.143	0.001
Relative biological aging index	4.43	9.36	−0.60	10.09	3.984	0.001
Psychological age	37.12	15.46	46.98	17.52	−4.563	0.001
Psychobiological age maturity	1.03	0.546	1.18	0.58	−2.005	0.05
Relative psychological aging index	9.60	15.09	5.19	16.52	2.142	0.05
Calendar age	27.53	4.61	41.77	5.61	−20.891	0.001

**Table 3:** Regression analysis (by the method of step-by-step inclusion) of the relationship between religious indicators and indicators of biopsychological age in a professional.

Sample 295 People			
Dependent Variable	Indicators	Values	Std. Error
Biological age	Multiple determination coefficient R2	0.044	
	Constant	42.411	2.478
	*Factors (predictors)*	*Non-standardized coefficient B/standardized Beta coefficient*	
	Asthenic experiences	0.361 ***/0.311 ***	0.100
	Pre-religious experiences	−0.246 */−0.180 *	0.108
	Spirituality	−0.083 */−0.131 *	0.042
Self-assessment of health	Multiple determination coefficient R2	0.070	
	Constant	3.261	1.096
	*Factors (predictors)*	*Non-standardized coefficient B/standardized Beta coefficient*	
	Pre-religious experiences	0.251 ***/0.348 ***	0.054
	The general indicator of religious experiences	−0.037 **/−0,204 **	0.013
Psychological age	Multiple determination coefficient R2	0.041	
	Constant	31.618	2.561
	*Factors (predictors)*	*Non-standardized coefficient B/standardized Beta coefficient*	
	Sthenic experiences	0.329 ***/0.202 ***	0.093
Psychobiological age maturity	Multiple determination coefficient R2	0.020	
	Constant	0.841	0.100
	*Factors (predictors)*	*Non-standardized coefficient B/standardized Beta coefficient*	
	Spirituality	0.005 **/0.143 **	0.002
Relative psychological aging index	Multiple determination coefficient R2	0.014	
	Constant	4.311	2.167
	*Factors (predictors)*	*Non-standardized coefficient B/standardized Beta coefficient*	
	The general indicator of religious experiences	0.060 */0.118 *	0.030

Note. * *p* < 0.05, ** *p* < 0.01, *** *p* < 0.001.

## References

[1] Berezina T. (2020). Differences in individual life path choices affecting life expectancy and health in Russia. E3S Web of Conferences.

[2] Koteneva A. (2020). The life-meaning orientations and biopsychological age of correctional officials. Psychol. Law.

[3] Koteneva A.V. (2020). Psychological factors of biopsychological age of law enforcement personnel. Agathos.

[4] Krause H., Hayward D., George L.K., Ferraro K.F. (2016). Religion, Health, and Aging. Handbook of Aging and the Social Sciences.

[5] Mirucka B., Bielecka U., Kisielewska M. (2016). Positive orientation, self-esteem, and satisfaction with life in the context of subjective age in older adults. Pers. Individ. Differ..

[6] Papadopoulos D. (2020). The Role of Well-Being, Spirituality, and Religiosity for Successful Aging in Late Life: A Brief Review. Adv. Aging Res..

[7] Zimmer Z., Jagger C., Chiu C.-T., Ofstedal M.B., Rojo F., Saito Y. (2016). Spirituality, religiosity, aging and health in global perspective: A review. SSM Popul. Health.

[8] Koteneva A. (2020). Personal health in the context of Christian worldview. Agathos.

[9] Shankov F.M. (2015). Religious and Spiritual Coping. An Overview of the Western Studies. Couns. Psychol. Psychother..

[10] Koteneva A.V., Chelyshev P.V. (2020). Spiritual and moral bases of psychological safety of mining-students. Eurasian Min..

[11] Berezina T.N., Rybtsova N.N., Rybtsov S.A. (2020). Comparative Dynamics of Individual Ageing among the Investigative Type of Professionals Living in Russia and Russian Migrants to the EU Countries. Eur. J. Investig. Health Psychol. Educ..

[12] Berezina T., Rybtsov S. (2021). Acceleration of Biological Aging and Underestimation of Subjective Age Are Risk Factors for Severe COVID-19. Biomedicines.

[13] Huguelet P., Koenig H.G. (2009). Religion and Spirituality in Psychiatry.

[14] Tuncay T. (2007). Spirituality in coping with HIV/AIDS. HIV AIDS Rev..

[15] Carmody J., Reed G., Kristeller J., Merriam P. (2008). Mindfulness, spirituality, and health-related symptoms. J. Psychosom. Res..

[16] Sharma V., Marin D.B., Koenig H.K., Feder A., Iacoviello B.M., Southwick S.M., Pietrzak R.H. (2017). Religion, spirituality, and mental health of U.S. military veterans: Results from the National Health and Resilience in Veterans Study. J. Affect. Disord..

[17] Koenig H.G., Al Zaben F., Khalifa D.A. (2012). Religion, spirituality and mental health in the West and the Middle East. Asian J. Psychiatry.

[18] Koenig H.G., Al Zaben F.N., Al Shohaib S., Wright J.D. (2015). Religion and Health: Clinical Considerations and Applications: International Encyclopedia of the Social & Behavioral Sciences.

[19] Rippentrop E.A., Altmaier E.M., Chen J.J., Found E.M., Keffala V. (2005). The relationship between religion/spirituality and physical health, mental health, and pain in a chronic pain population. Pain.

[20] Makhnach A.V. (2016). Resilience Capacity of Person and Family: Social and Psychological Paradigm.

[21] Chelyshev P.V., Koteneva A.V. (2019). Personal factors of mining students resilience. Min. J..

[22] Smirnov D.O. (1999). The description of the standardization procedures of psychometric techniques—«Questionnaire religious activity»: Paschi. Sci. Psychol. J..

[23] Markina L.D. (2001). Determination of the Biological Age of a Person by V.P. Voitenko Method.

[24] Berezina T. (2020). Distribution of biomarkers of aging in people with different personality types. E3S Web Conf..

[25] Voitenko V.P., Tokar A.V. (1983). The assessment of biological age and sex differences of human aging. Exp. Aging Res..

[26] Barak B. (2009). Age identity: A cross-cultural global approach. Int. J. Behav. Dev..

[27] Jianbin X., Mehta K.K. (2003). The effects of religion on subjective aging in Singapore: An interreligious comparison. J. Aging Stud..

